# Efficacy of Virtual Reality Exposure Therapy in the Treatment of PTSD: A Systematic Review

**DOI:** 10.1371/journal.pone.0048469

**Published:** 2012-12-27

**Authors:** Raquel Gonçalves, Ana Lúcia Pedrozo, Evandro Silva Freire Coutinho, Ivan Figueira, Paula Ventura

**Affiliations:** 1 Institute of Pschology, Universidade Federal do Rio de Janeiro, Rio de Janeiro, Brazil; 2 Department of Epidemiology, Escola Nacional de Saúde Pública (ENSP-FIOCRUZ), Rio de Janeiro, Brazil; 3 Institute of Psychiatry, Universidade Federal do Rio de Janeiro, Rio de Janeiro, RJ, Brazil; ICREA-University of Barcelona, Spain

## Abstract

The use of Information and Communication Technologies, such as virtual reality, has been employed in the treatment of anxiety disorders with the goal of augmenting exposure treatment, which is already considered to be the first-line treatment for Post-traumatic Stress Disorder (PTSD). To evaluate the efficacy of virtual reality exposure therapy (VRET) in the treatment of PTSD, we performed a systematic review of published articles using the following electronic databases: Web of Science, PubMed, PsycINFO, and PILOTS. Eligibility criteria included the use of patients diagnosed with PTSD according to DSM-IV, the use of cognitive behavioral therapy (CBT) and the use of virtual reality for performing exposure. 10 articles were selected, seven of which showed that VRET produced statistically significant results in comparison to the waiting list. However, no difference was found between VRET and exposure treatment. Of these 10, four were randomized, two were controlled but not randomized and four were non-controlled. The majority of the articles used head-mounted display virtual reality (VR) equipment and VR systems specific for the population that was being treated. Dropout rates do not seem to be lower than in traditional exposure treatment. However, there are a few limitations. Because this is a new field of research, there are few studies in the literature. There is also a need to standardize the number of sessions used. The randomized studies were analyzed to assess the quality of the methodology, and important deficiencies were noted, such as the non-use of intent-to- treat-analysis and the absence of description of possible concomitant treatments and comorbidities. Preliminary data suggest that VRET is as efficacious as traditional exposure treatment and can be especially useful in the treatment of patients who are resistant to traditional exposure.

## Introduction

Post-traumatic stress disorder (PTSD) involves a constant feeling of fear generated by inadequate consolidation of the autobiographical trauma memory [1]. Foa & Kozak [2] suggested that for there to be adequate processing of the traumatic memory and the consequent extinction of the fear, the memory must be activated and safe components must be inserted. Prolonged Exposure Therapy, proved to be highly eficacious in the treatment of PTSD, aims to access the traumatic memory, including information about the traumatic situation and related emotions, thoughts and behaviors. It helps the patient to understand the context of the traumatic experience as well as its impact in the patient's life. It also enables the patient to achieve a realistic perspective on the traumatic event and its aftermath [3].

Despite the fact that exposure therapy stimulates emotional engagement during imaginal exposure, some patients find it difficult to immerse themselves in the traumatic scene and, therefore, may quit the treatment. In some studies, dropouts and non-response rates may reach 50% of the cases [4]. Therefore, the use of Information and Communication Technologies may facilitate exposure for avoidant patients.

In this sense, virtual reality has been used as a tool for exposure and has achieved positive results in the treatment of various anxiety disorders including specific phobias, social phobia, panic disorder and PTSD [5]. Even though this resource has encountered some difficulties because it raises questions about affecting the therapeutic relationship and struggles with personalizing exposure for individuals with different traumas [5], its use has yielded many benefits. Virtual reality exposure therapy (VRET) facilitates the emotional engagement of patients with PTSD during exposures to the multiple sensory stimuli made possible by the virtual environment, bypassing symptoms of avoidance and facilitating control on the part of the therapist [6]–[7]. The sense of presence provided by a virtual environment that is rich in sensory stimuli facilitates the emotional processing of memories related to the trauma [8]. This technological apparatus allows gradual exposure to the feared environment according to the needs of each patient. In addition, it can be used in situations where time is limited, as well as in situations that are difficult to control or unpredictable [9] or that could put the patient at risk if the exposure were performed in a real situation. Finally, exposure in a virtual reality environment allows for greater methodological rigor in clinical studies as it allows for the standardization of the duration and type of exposure for all patients [10].

The objective of this article is to conduct a systematic review of studies that have used virtual reality in the treatment of PTSD. We aim to verify the efficacy of Virtual Reality Exposure Therapy for patients diagnosed with PTSD.

## Methodology

We performed an electronic search in the following databases: ISI/Web of Knowledge, PUBMED/MEDLINE, PILOTS and PsycINFO in May 2011 with keywords that included the terms PTSD OR “stress disorder*” and “virtual reality”. In the ISI/Web of Knowledge, we restricted the search criteria to include only “articles” and “notes”. Articles found in the references of the articles selected for this review were also considered for analysis. Independently data collection process was applied. The articles included in the final selection had to fulfill the following criteria.

Criteria for inclusion: the use of patients diagnosed with PTSD according to DSM-IV, the use of cognitive-behavioral therapy (CBT) and the use of virtual reality for performing exposure.

Criteria for exclusion: theoretical articles, reviews, theses, dissertations and book chapters, articles not published in a peer-reviewed journal, articles that used patients who were not diagnosed with full PTSD or techniques that were not part of the repertoire of CBT, case studies, articles that did not include outcome criteria and trials published in languages other than English, Portuguese or Spanish.

After the search phase, we did an analysis of the methodological quality of the randomized controlled trials based on the Cochrane Collaboration Tool for Assessing the Risk of Bias [11]. This tool analyzes the risk of bias for each study that might interfere with the results. In addition, six items were added that also represent risks for biased results. These items were based on the scale developed by Kocsis et al [12] and include description of relevant comorbidities; information on screened, included and excluded subjects; description of treatment (or reference); description of concurrent treatment; an intent-to-treat analysis; and description of dropouts.

## Results

**Figure 1:**
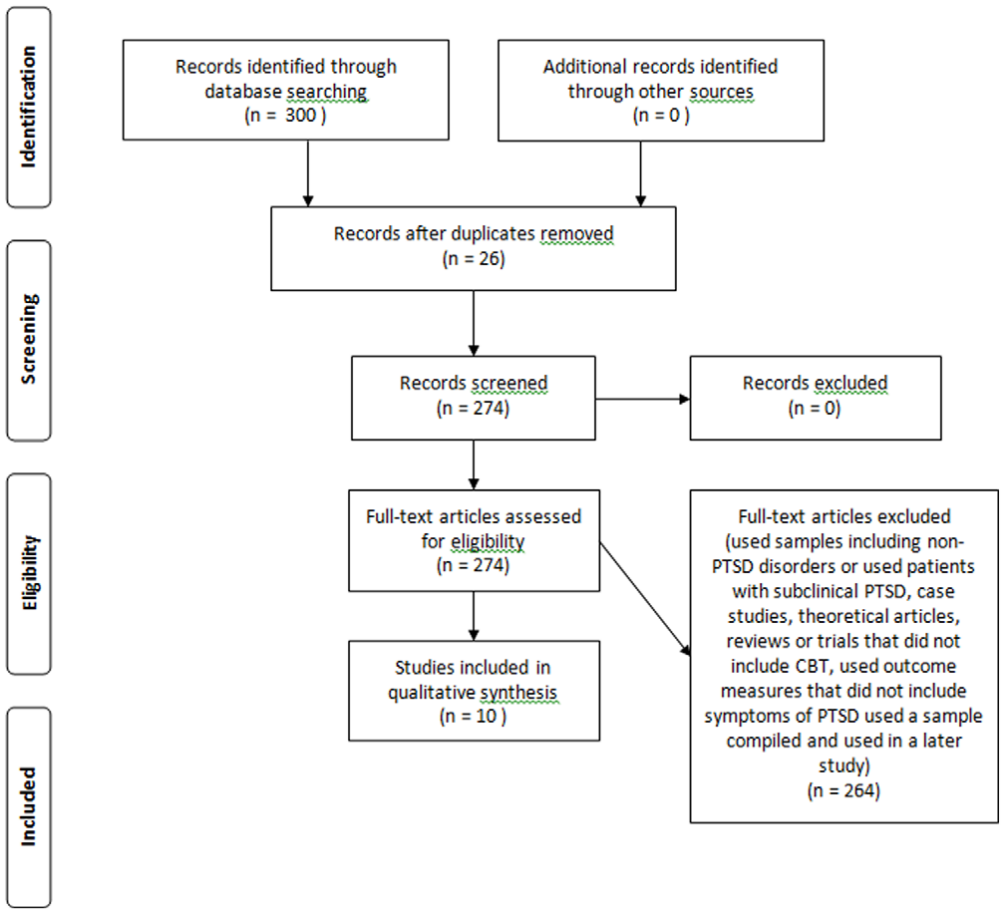
Flowchart of the process of identifying and selecting studies.

The final result of the search can be observed in the flowchart (Figure 1).

Of the 300 articles obtained through the electronic search summed from all the databases searched, 27 were excluded for using samples including non-PTSD disorders or using patients with subclinical PTSD, 36 were excluded for being case studies, 192 were excluded for being theoretical articles, reviews or trials that did not include CBT, two were excluded for using outcome measures that did not include symptoms of PTSD and seven were excluded for using a sample compiled and used in a later study. Thus, 10 articles were considered for the final analysis. This count includes articles found in more than one electronic database, which were, therefore, repeated. Additionally, some studies met more than one exclusion criterion. Each of the studies selected is described below.

Ready et al [7] performed an open trial with 21 Vietnam veterans. The final result combined the samples with those from an earlier study by Rothbaum et al [8], that consisted of a sample of five participants. Nine patients in the most recent study completed the treatment, and six patients dropped out during it. Of the 21 initial participants, 14 completed at least one post-treatment assessment. The questionnaires used included the Clinician Administered PTSD Scale (CAPS), Impact of Events Scale (IES), Beck Depression Inventory (BDI) and Subjective Units of Discomfort Scale (SUDS). The protocol included 8 to 20 weekly sessions, and each lasted 90 minutes. The techniques used included psychoeducation, anxiety management techniques, exposure using VR and imaginal exposure. Follow-ups were performed at three and six months. There was a significant reduction in total CAPS and IES after treatment and during the follow ups. However, for the intrusion cluster on the CAPS scale, no statistical significance was observed at the first measure post-treatment; however, at the follow-ups, there was significant improvement compared to the beginning of treatment. Although there was a significant reduction in BDI during post-treatment and at the follow-up at six months, this was not evident at the three-month follow-up. Gains were maintained for 10 of the eleven patients who completed all of the follow-ups.

Difede et al [13] evaluated the efficacy of treatment with VRET in-patients who developed PTSD after the terrorist attack on the World Trade Center. The participants were assigned to two groups. In the first group, thirteen patients underwent intervention with VRET, and eight matched subjects were assigned to the waiting list. Five of the thirteen participants in the first group were resistant to previous treatments, and four were resistant to imaginal treatment. Because it was decided that these patients would be assigned to treatment, the study became quasi-experimental. The participants in the VRET group were paired with the participants on the waiting list according to the severity of their CAPS scores and socio-demographic characteristics. The study sample was composed entirely of males with severe PTSD. In addition to the use of CAPS, the participants were evaluated by an independent assessor using BDI and Clinical Global Impression (CGI). The treatment combined CBT with VR using techniques including psychoeducation, relaxation training, cognitive restructuring and VRET. The number of sessions was less than 14, with a mean of 7.5. All of the patients received at least six weeks of VRET. The results showed that nine of the 10 participants who completed the training obtained clinically and statistically significant improvements. A large effect size was obtained (Cohen's d = 1.54). Compared to the waiting list, the group treated with VRET demonstrated statistically significant improvement based on the CAPS score. The same did not occur for BDI and CGI, for which an interaction between time and group was not noticed. However, the authors stated that the baseline scores were low. At the end of the study, six of the 10 participants in the VRET group no longer met the diagnostic criteria for PTSD. Three patients from the group undergoing VRET did not complete the treatment, and one dropped out due to the anxiety related to the exposure. The dropout rate in the control group was not reported. The results remained the same in the follow-up at six months.

Wood et al [14] examined the effects of VRET on twelve participants in a non-controlled study. 10 sessions that lasted 90 to 100 minutes each were performed once or twice a week. The Post Traumatic Stress Disorder Checklist (PCL-M), the BAI and PHQ-9 questionnaires were used. The techniques and procedures used in the study included psychoeducation, meditation for anxiety management, cognitive restructuring, biofeedback training and gradual exposure using virtual reality. There was a significant reduction in the PCL in 75% of the participants, and these cases no longer fulfilled the criteria for PTSD. The study did not report if there were dropouts over the course of the treatment, as well as averages or standard-deviations and only showed the results with a graph. The study did not perform a follow-up. The focus of this article was on the cost-benefit analysis of using VR; thus, the article may be limited in terms of methodology.

A randomized study by Ready et al [15] compared VRET with Therapy Centered on the Present (TCP) in patients who are veterans of the Vietnam War. Of eleven male participants, nine completed the treatment, with five in the VRET group and four in the TCP group. Protocols for both treatments consisted of 10 sessions. The CAPS and BDI scales were administered before and after treatment and during a follow-up six months later by an independent assessor who was unaware of the treatment. There was an improvement of symptoms in both conditions from pretreatment to post-treatment and at the follow-up, performed six months after the end of the study, with scores on the CAPS scale showing greater variations for VRET. However, there was not a statistically significant improvement on the CAPS and BDI scales either within or between groups. It was not possible to compare and find statistically significant differences due to the small sample. There were two dropouts, one from each group.

Roy et al [16] studied soldiers diagnosed with PTSD using functional magnetic resonance imaging (fMRI) in addition to evaluating the effects of VRET. In the first phase of the study, there were 29 subjects who were assigned to four groups: fifteen with both PTSD and Traumatic Brain Injury (TBI), nine with PTSD only, one with TBI only, and four controls. The fMRI was performed before and after treatment and during follow-up periods. In the treatment phase, fifteen participants with PTSD were initially selected at random for VRET (seven) or for Prolonged Exposure (eight). At least twelve sessions lasting 90 min were performed over ≥ six weeks. Both the PE and VRET treatments used psychoeducation, relaxation techniques and some elements of imaginal and in vivo exposure. The groups received equivalent times of exposure (with VR or imaginal exposure), which lasted up to half of each session. The CAPS, PCL-M and BDI scales were administered at the same time as the cerebral scans. It was an ongoing study in which eight participants with PTSD had completed the final scan, and six had dropped out. As the authors considered the number of participants in each group insufficient for a comparison of the efficacy of treatments, the results of the two groups were combined, meaning, in this respect, that the study was non-controlled. There was a non-significant improvement in the clinical symptoms, which could be measured through reductions in CAPS, PCL, CGI and BDI. The results were maintained in the follow-ups performed at one, two and three months with eight patients with PTSD completing the post-treatment scan (five with both PTSD and TBI and three with PTSD only). The brain regions of interest were the amygdala, the hippocampus and the anterior cingulate cortex. Significant improvements are evident on the fMRI scans, which showed a reduced activation of the amygdala, subcallosal gyrus and lateral prefrontal cortex as well as a less-accentuated reduction for the anterior cingulate cortex. These results are corroborated by CGI scores; however, the CAPS score improvements are not statistically significant.

The study by McLay et al [17] is a controlled non-randomized study that shows the results of treatment for PTSD in soldiers from the Iraq War, all male. They compared six cases treated with VRET and four cases with traditional exposure therapy. The treatments were conducted once or twice per week, and the number of sessions varied between six and twelve. There was no standardization in the administration of the techniques or in the number of sessions. The patients were treated according to the clinical judgment of what they required, and they typically received the treatment to which they were assigned. The scales administered included PCL-M, CAPS, PHQ-9 and BAI. All of the patients who were treated with VRET significantly reduced their scores on the PCL-M (by 67% on average), BAI and PHQ-9. At the end of the treatment, five no longer met the diagnostic criteria for PTSD. The patients who received traditional exposure therapy also showed statistically significant improvement at the end of the treatment (a 74% decrease on the PCL-M), and none showed the diagnostic criteria for PTSD at this stage. There was no statistically significant difference between the treatments. The study did'nt perform a follow up. There were no dropouts.

Rizzo et al [18] conducted an open trial with 40 war veterans who were resistant to pharmacological treatment and counseling using virtual Iraq. Sessions lasting 90 to 120 minutes were held twice per week for five weeks. The average number of sessions was less than 11. The techniques used included psychoeducation, anxiety management techniques and imaginal, in vivo and VR exposure. Participants were evaluated based on the PCL-M, BAI and Patient Health Questionnaire (PHQ-9), in addition to physiological monitoring, which included heart rate, galvanic skin response and respiration frequency. However, the results of the psychophysiological monitoring were not reported. A follow-up was performed at three months. Of the 20 participants who completed the treatment, there was a clinical and statistically significant reduction in the PCL-M, with 17 participants showing a reduction of more than 50%. Furthermore, sixteen of the participants did not fulfill the criteria for PTSD after treatment. When looking at the average scores for the BAI and the PHQ-9, there was a statistically significant reduction at the end of treatment. Half of the patients did not complete the treatment.

Seven soldiers dropped out before the first session, six quit after the first session, and seven at the beginning of the VR treatment. Gains were maintained at follow-ups.

The study by Botella et al [19] included subjects who had experienced a broad variety of traumatic events and used EMMA's World as a VR tool. This system used symbols to represent various traumatic events. Their sample included eight men and two women. This was a randomized clinical trial with two groups that used VRET or CBT with in vivo and imaginal exposure. Participants on the waiting list were not included. The study included 10 participants. The authors did not report the number of participants in each group or whether any participant did not complete treatment. Both protocols used from nine to twelve sessions. There was a statistically significant reduction on all the scales administered (CAPS, Davidson Trauma Scale (DTS) and Posttraumatic Cognitions Inventory (PTCI) between pre- and post-treatment conditions for the group that received VRET. VRET proved to be more effective than traditional therapy on the CAPS scale, but the difference was not statistically significant for the CAPS scale or for the others administered. The researchers did not perform follow-ups and did not report the dropout rate for either of the groups.

The study by Gamito et al [20] evaluated VRET as an alternative procedure for reducing symptoms of chronic PTSD in elderly Portuguese veterans who had participated in the colonial war in Africa. 10 participants, all male, were randomly assigned to three groups: five for VRET, two for imaginal exposure, and three for the waiting list. The protocols for VRET and imaginal exposure were based on cognitive desensitization with twelve sessions of exposure with virtual reality for VRET and twelve sessions of traditional imaginal exposure for imaginal exposure group. The first session for both protocols was dedicated to psychoeducation. The CAPS, Impact of Events Scale Revised (IES-R), Symptoms Checklist Revised (SCL-90-R) and BDI scales (only for the VR group) were administered before and after treatment. There was no statistically significant difference in the analysis of the CAPS scale. However, the group treated with VRET obtained a reduction of 8% when measured with CAPS, although there was only a reduction of 1% for the group assigned to imaginal exposure and 6% for the WL. There was a reduction in IES-R for the group with VRET, whereas an increase was observed for the other two groups. There was a significant reduction in symptoms of depression (reduction of 40% on the BDI) and on the SCL-90-R for the VRET group. No follow-up was performed. One dropout was reported in the VRET group, and the dropout rate in the control group was not reported.

The study by McLay et al [21] evaluated the efficacy of VRET in the treatment of 20 Active Duty military personnel in a randomized trial. 10 of these patients were assigned to VRET and 10 to treatment as usual (TAU). The participants of the latter group received a combination of typical treatments for PTSD, which included Prolonged Exposure, Cognitive Processing Therapy, Eye Movement Desensitization and Reprocessing, group therapy, psychiatric medication, substance rehabilitation and inpatient service. The VRET protocol included sessions of psychoeducation, relaxation, attentional and autonomic control training and exposure to a VR simulation of Iraq or Afghanistan. There was no standardization of the treatment protocol, and the number of sessions varied from 10 to 13 weekly sessions for the two groups. The participants were monitored using the CAPS scale before and after treatment. At the end of the treatment, seven of the 10 participants in the VRET protocol showed improvements of greater than 30% on the CAPS scale, whereas only one of the nine participants who completed the TAU showed an improvement greater than 30%. Both treatments showed efficacy and there was no statistically significant difference between the groups. Only one dropout in the TAU group was reported. The researchers did not perform a follow-up.

**Figure 2:**
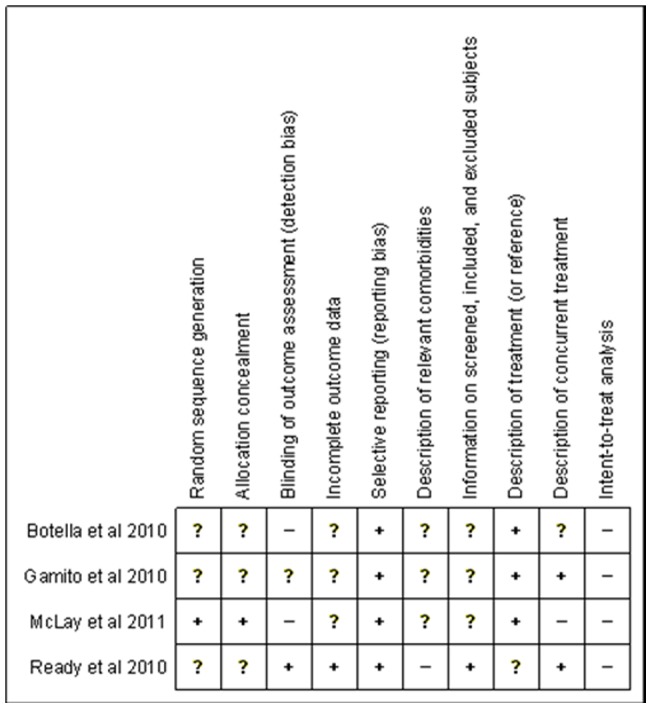
Methodological Analysis of Randomized Controlled Trials; + Low Risk of Bias; – High Risk of Bias; ? Unclear Risk of Bias.

**Figure 3:**
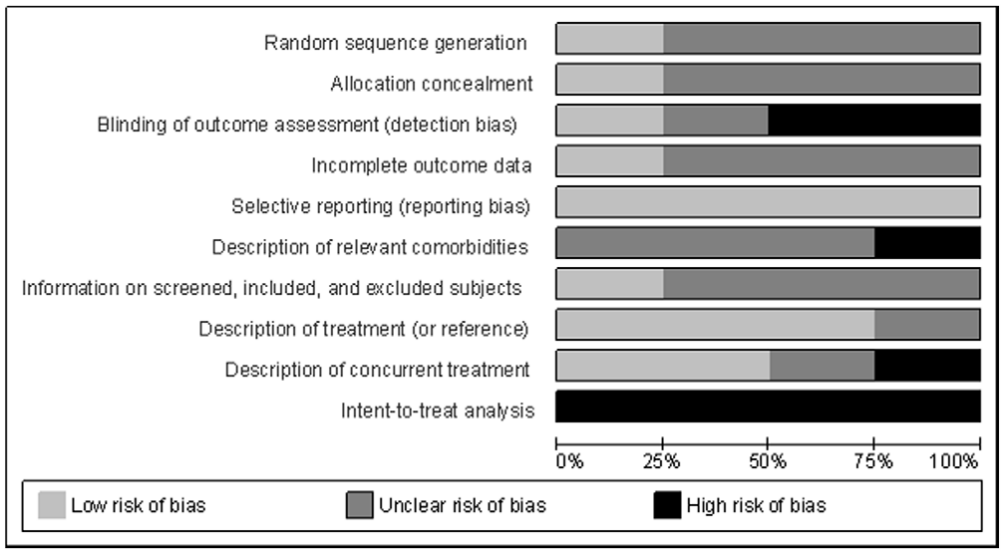
Methodological Analysis of Randomized Controlled Trials.

Figures 2 and 3 summarize the different aspects concerning the methodological quality of the randomized studies. Among these investigations, only one study reported the method used for generating the random sequence and whether the method used to hide the sorting did not allow a prediction of the distribution of the patients into groups. Two of the studies did not use a blind evaluator; one did not report on this, and one made use of a blind evaluator. In addition, only one of the studies included data for all the subjects in the final analysis. In all of the articles, the main scales were also administered at the final evaluation. In one study, the presence of comorbidities was not described, and in three studies, they were partially described. The numbers of subjects screened, included and excluded were described completely in only one study. In three studies, the treatments were sufficiently described or referenced to allow replication. Only two studies clearly provided information on concurrent treatments allowed and administered during the course of the study, and they did not permit concomitant treatments. In one study, this description was partially completed, and in another study, there was the possibility that the participants in group therapy were concurrently taking non-stabilized psychotropic medication. None of the articles performed an intent-to-treat analysis because there were no evaluations over the course of treatment. In fact, there were only pre- and post-treatment evaluations.

## Discussion

### General results

To the best of our knowledge and despite the current relevance of this subject, this is the first systematic review that evaluates the efficacy of VRET focused exclusively on patients diagnosed with PTSD. A study conducted by Meyerbroker & Emmelkamp [5] included a systematic review of VRET in various anxiety disorders including PTSD. However, only two studies were considered in their analysis [7]–[13]. A meta-analysis was performed by Parsons et al [22], and two studies with PTSD were selected. The meta-analysis was based on fewer than 20 subjects. Two different meta-analyses in VRET and anxiety disorders were published [23]–[24], including only one paper in PTSD [13]. The lack of systematic reviews and meta-analyses on the subject is probably due to the small number of studies in the literature that use VR as a tool for exposure. However, the few published studies are recent, with the first dating from 2006 [7], which indicates rapid growth in this area.

Based on the evaluations of the 10 studies, the results point to the potential efficacy of VRET in the treatment of PTSD. Among the six studies that included a control group, a statistically significant reduction in questionnaires evaluating symptoms of PTSD was observed in four. In comparison to the waiting list, results were significantly better for VRET. However, no difference was found between VRET and exposure treatment. The same result was obtained in the meta-analyses on the efficacy of VRET and classical cognitive-behavioral evidence-based treatment in the treatment of anxiety disorders, performed by Opris & colleagues [24]. We cannot discard, nonetheless, the possibility of a Type 2 error due to low sampling power; that is, if the samples were larger, a difference in efficacy in favor of one of the treatments might have been found. In three of the four non-controlled studies, VRET showed efficacy, which confirms the potential efficacy of this treatment.

Eight of the 10 studies used samples of war veterans. The high utilization of this population was probably due to the high financial and social costs of wars in terms of the number of soldiers who return home with PTSD [14]. In two studies, samples included victims of various traumas, and the remaining study was conducted with victims of the terrorist attack of September 11, 2001. War veterans are considered to be a more resistant sample^7^. Thus, the results obtained might have been even more promising if the studies had used subjects exposed to other types of trauma. A problem involved in using VRET in comparison to traditional exposure is related to the difficulty of constructing a context that refers precisely to the traumatic memory of the patient [5]. Due to its high financial cost, it would be almost impossible to follow the idiosyncratic method, which means personalizing the virtual environment according to the perspective of each patient related to the trauma. By contrast, with a homogeneous sample, only some stimuli elicited by the virtual environment are necessary to produce anxiety sufficient for activation of the traumatic memory [7]. In a study which used individuals that had suffered various types of trauma [19], the virtual environment was even more general, but it still evoked recollections of the traumatic event.

The number of sessions varied between 5 and 20. Only four studies set a fixed number of sessions. The others proposed only a minimum number that could be exceeded depending on the needs of the patient. The lack of standardization makes it difficult to evaluate the efficacy of the protocol, although it is understandable considering that these were the first studies to test the efficacy of VRET in the treatment of PTSD. Therefore, there is no standard to follow. The sessions basically consisted of techniques that included psychoeducation, anxiety management (such as diaphragmatic breathing, progressive muscle relaxation and meditation) and *in vivo* and imaginal exposure. Only three studies reported using cognitive restructuring [13], [14]–[21].

The majority of the articles used head-mounted display VR equipment and VR systems specific for the samples. Only one study used an automatic virtual environment, an adaptive VR display called EMMA's World [19]. This system allows for deep immersion into the virtual environment for both the patient and the therapist, which offers a better perception of what the patient is seeing. This VR equipment is non-specific, and different environments could bring the patient into contact with different feelings that serve as triggers for traumatic memories. No other studies used the automatic virtual environment methodology. So, it was not possible to test which of the two methodologies was more efficacious in the treatment of PTSD.

### Methodological Issues

#### Randomized clinical trials

We found that the four randomized studies were not conducted in accordance with rigorous criteria for quality. All of them had methodological limitations that introduced a risk of bias for the final results. Proper reporting of the items that constitute a randomized trial assists in the critical evaluation of internal validity and the generalization of the results [25].

It is striking the absence of information to judge the quality of the trials. The majority of studies omitted data concerning random sequence generation, allocation concealment, description of relevant comorbidities, information on screened, included and excluded subjects and description of incomplete outcome data. Besides, even in the study with the higher amount of items evaluated favorably [15], those items account for less than half of the total of the evaluated items. The items with the lower risk of bias were selective reporting and description of treatment.

Another serious limitation concerns the use of extremely small samples in each of the randomized trials. For example, two studies [19]–[20], despite being randomized, involved only 10 participants each. The randomized study with the largest sample included 20 subjects [21]. These small samples make a meta-analysis focused on those data impossible. Finally, the absence of effect size makes it difficult to understand to what extent the result is explained by the predictor variable [26]. In addition, none of the studies used the number needed to treat (NTT), which is a more powerful and intelligible statistical tool because it establishes the necessary minimum number of individuals to be treated in the event that one of them no longer fulfills the diagnostic criteria after the treatment [27].

Future studies should take into account the methodological issues mentioned above and presented in figures 2 and 3 in order to improve the quality of the trials in VRET.

#### All Studies

We observed an absence of key information that is required to understand the evaluation of VRET efficacy because some studies focused on the description of the VR system configured for exposures, thereby placing less emphasis on evaluating the efficacy of the treatment. Some studies did not even present quantitative results but only qualitative information about efficacy [14]–[18].

In spite of these limitations, all of the articles reported the results of the most important scale at the final evaluation. Therefore, there was no risk of hidden possible negative results. In addition, half of the articles performed follow-ups. Those data were collected from one month to one year after the end of treatment. Although the ideal time suggested for a follow-up begins at twelve months [12], the fact that there was follow-up assessment shows an effort to incorporate a more robust methodology. We believe that, with advances in the study of VRET efficacy in the treatment of PTSD, future studies will achieve better methodology quality.

Another important consideration is treatment adherence. It is known that exposure techniques can have a high rate of non-adherence due to the initial worsening of the symptoms. For there to be extinction of the fear associated with the memory of the traumatic event, it is necessary for the patient to experience anxiety and feel its reduction after a specified period of repetition of the traumatic memory. Because the technique is highly anxiogenic, many patients do not agree to undergo the treatment or give up at the beginning of treatment. Theoretically, VRET appears to partially bypass this problem by providing exposure in a more controlled and safer environment. However, except for two studies [17]–[21], there were dropouts from the VRET group for all of the studies that supplied this information. Two studies did not report the dropout rate [14]–[19]. The highest dropout rate occurred in the study by Rizzo et al [18], where half of the total sample of 40 participants did not complete the treatment. Two other studies showed high dropout rates [16]–[7], with six dropouts in each (out of 21 and 29, respectively).

It is important to mention the low treatment adherence rates found in the active duty military populations. Out of 49,425 veterans diagnosed with PTSD, only 9.5% attended nine or more VA mental health sessions in 15 weeks or less in the first year of diagnosis [28]. This population has issues regarding stigma associated with mental health problems, higher rates of alcohol related problems, and competing responsibilities for preparing for the next deployment that have a dramatic negative effect on adherence to treatment [29]–[30].

Despite the fact that exposure therapy is considered to be highly aversive [31], in a study by Hembree et al [32], the dropout rates for exposure therapy were not different from the rates found for techniques considered to be less aversive, such as Cognitive Therapy, Stress Inoculation Training and EMDR during the treatment of PTSD. However, due to the high dropout rate that was evident in the selected articles, we believe that there remains a certain loss of control of anxiogenic stimuli during VRET. The loss of control might be related to the need for the therapist or the patient himself to manage the equipment so as to reduce the intensity of the stimulus when anxiety is very high. The use of new technologies, such as physiology-driven adaptive VR stimulation that automatizes the system to make it less dependent on the intervention of the psychotherapist during exposure, may be able to bypass this limitation.

Finally, one potential application of VRET would be for patients who are resistant to traditional exposure. For example, Difede et al [13] included five patients who had not responded initially to traditional exposure, and the results showed a positive response to VRET (for three of them, there was a reduction of at least 25% in CAPS, whereas for the other two, the reduction was greater than 50%). Replicating this finding would provide evidence of the mechanisms by which VRET could be more advantageous than traditional exposure. We believe that the results would be promising because VRET prevents patients from using safety signals that make engagement difficult, which compromises the adequate extinction of conditioned fear.

### Conclusions

The results of this systematic review suggest the potential efficacy of VRET in the treatment of PTSD for different types of trauma. VRET proved to be as efficacious as exposure therapy. VRET can be particularly useful in the treatment of PTSD that is resistant to traditional exposure because it allows for greater engagement by the patient and, consequently, greater activation of the traumatic memory, which is necessary for the extinction of the conditioned fear.

However, there remains a vast field to be explored that requires methodologically stronger trials and replications of those trials, the standardization of treatment, empirical studies with different VR systems and attempts to increase the rate of adherence to treatment. In future studies, the use of VRET for traditional exposure resistant patients would be interesting, as would the use of artificial intelligence to make the system less dependent on the intervention of the psychotherapist at the moment of exposure.

## Supporting Information

Checklist S1 **PRISMA 2009 Checklist.**
(DOC)
